# Preliminary results of a novel sphincter-sparing technique—fistula occlusion with the internal sphincter flap (FOISF)—for high complex anal fistulas

**DOI:** 10.1093/gastro/goaf006

**Published:** 2025-01-18

**Authors:** Dandan Huang, Ning Wang, Yiping Li, Donglin Ren, Yong Yang, Kaikai Wei, Yanzhu Li, Zhimin Liu

**Affiliations:** Department of General Surgery (Coloproctology), The Sixth Affiliated Hospital, Sun Yat-sen University, Guangzhou, Guangdong, P. R. China; Guangdong Provincial Key Laboratory of Colorectal and Pelvic Floor Diseases, The Sixth Affiliated Hospital, Sun Yat-sen University, Guangzhou, Guangdong, P. R. China; Biomedical Innovation Center, The Sixth Affiliated Hospital, Sun Yat-sen University, Guangzhou, Guangdong, P. R. China; Department of Radiology, The Sixth Affiliated Hospital, Sun Yat-sen University, Guangzhou, Guangdong, P. R. China; Department of General Surgery (Coloproctology), The Sixth Affiliated Hospital, Sun Yat-sen University, Guangzhou, Guangdong, P. R. China; Guangdong Provincial Key Laboratory of Colorectal and Pelvic Floor Diseases, The Sixth Affiliated Hospital, Sun Yat-sen University, Guangzhou, Guangdong, P. R. China; Biomedical Innovation Center, The Sixth Affiliated Hospital, Sun Yat-sen University, Guangzhou, Guangdong, P. R. China; Department of General Surgery (Coloproctology), The Sixth Affiliated Hospital, Sun Yat-sen University, Guangzhou, Guangdong, P. R. China; Guangdong Provincial Key Laboratory of Colorectal and Pelvic Floor Diseases, The Sixth Affiliated Hospital, Sun Yat-sen University, Guangzhou, Guangdong, P. R. China; Biomedical Innovation Center, The Sixth Affiliated Hospital, Sun Yat-sen University, Guangzhou, Guangdong, P. R. China; Anorectal Department, Xuzhou Central Hospital, Xuzhou, Jiangsu, P. R. China; Department of Radiology, The Sixth Affiliated Hospital, Sun Yat-sen University, Guangzhou, Guangdong, P. R. China; Department of Anorectal Surgery, The People’s Hospital of Zhongshan, Zhongshan, Guangdong, P. R. China; Department of General Surgery (Coloproctology), The Sixth Affiliated Hospital, Sun Yat-sen University, Guangzhou, Guangdong, P. R. China; Guangdong Provincial Key Laboratory of Colorectal and Pelvic Floor Diseases, The Sixth Affiliated Hospital, Sun Yat-sen University, Guangzhou, Guangdong, P. R. China; Biomedical Innovation Center, The Sixth Affiliated Hospital, Sun Yat-sen University, Guangzhou, Guangdong, P. R. China

**Keywords:** complex anal fistula, high transphincteric anal fistula, suprasphincteric anal fistula, internal sphincteric flap, sphincter-sparing approach

## Abstract

**Background and aim:**

High complex anal fistula is a clinical challenge for proctologists and a nightmare for patients. Although the sphincter-sparing approach seems an ideal surgical intervention, there remains room for improvement in treatment efficacy. Herein, we introduce an enhanced sphincter-sparing approach, namely the fistula occlusion with the internal sphincter flap (FOISF), for treating high complex anal fistulas.

**Methods:**

This study evaluated 15 patients with high complex anal fistulas who underwent FOISF between October 2021 and December 2022 in the Sixth Affiliated Hospital, Sun Yat-sen University (Guangzhou, P. R. China). Data on success rates, anal function, and various surgical characteristics were subjected to rigorous analysis.

**Results:**

All patients underwent the FOISF procedure, with a median operation time of 53 min. Fourteen patients achieved primary intention healing, while one patient healed by second intention. No recurrence was observed over a follow-up period of 14–30 months. All patients exhibited satisfactory anal continence, with no statistically significant difference observed between preoperative and postoperative Wexner scores (*P *=* *0.331). A significant improvement in the quality of life was observed when compared with the preoperative assessment (*P *<* *0.001).

**Conclusion:**

The preliminary results of the FOISF procedure present an effective approach to treat high complex anal fistula.

## Introduction 

Anal fistulas are a commonly encountered anorectal condition characterized by chronic infectious symptoms that can significantly diminish the patients’ quality of life. Surgical intervention for anal fistula remains the preferred approach. The primary objectives of reducing recurrence and preserving continence are of utmost importance and challenging to achieve [1]. Fistulotomy is a valuable technique for the effective treatment of uncomplicated low fistulas, with high success rates and maintenance of good continence. However, it is not suitable for high complex fistulas, as it can damage the anal sphincters and lead to impaired anal function [2]. Recently, several sphincter-sparing procedures have emerged and become popular among surgeons [3]. These procedures include techniques such as endorectal advancement flap (ERAF) [4], seton drainage [5], ligation of intersphincteric fistula tract (LIFT) [6], and transanal opening of intersphincteric space (TROPIS) [7]. However, current approaches have inherent limitations and factors that contribute to failure. ERAF is a strong technology-dependent procedure; however, laceration of mucosal flap is the most common reason for failure, making the repeat surgical cases more difficult. Modified seton drainage risks recurrence of the fistula after seton removal. The potential risks of the LIFT technique are failure to find and ligate the internal opening (IO) and undrained sepsis in the intersphincteric space, and the long-term result seems unreliable. TROPIS may not be suitable for cases with a bulky transphincteric tract or extensive extrasphincteric sepsis. Endofistular approaches, such as video-assisted anal fistula treatment and fistula-tract laser closure, have intrinsic shortage of IO management and show effectiveness in highly selective cases. Hence, further improvements are needed regarding sphincter-sparing surgery.

In this report, we introduce an enhanced sphincter-sparing approach for treating high transphincteric anal fistulas, which is modified and developed by our expert team. This method is an integration of different techniques, including resection of the IO, transanal suture of the transphincteric tract, and occlusion of the internal sphincteric flap. The detailed steps and preliminary results are presented.

## Patients and methods

### Patients

All consecutive patients with anal fistula between October 2021 and December 2022 who underwent fistula occlusion with the internal sphincter flap (FOISF) in the Sixth Affiliated Hospital, Sun Yat-sen University (Guangzhou, P. R. China), were included. The inclusion criteria were as follows: (i) age >16 years and <65 years and (ii) high transphincteric anal fistula (the tract crossing more than one-third of the external sphincter) or suprasphincteric anal fistula according to the Park’s classification [8]. The exclusion criteria were as follows: (i) perianal fistula secondary to Crohn’s disease, ulcerative colitis, tuberculosis infection, trauma, or malignant tumors and (ii) pregnancy, lactation, or history of serious mental illness. All the patients were operated on by Dr Liu and Dr Huang, both colorectal surgeons with >5 years’ experience in treating anal fistulas, especially complex anal fistulas. The study was reviewed and approved by the Ethics Committee of the Sixth Affiliated Hospital of Sun Yat-Sen University (approval number 2023ZSLYEC-605).

### Preparations and operative procedure

**Figure 1.**
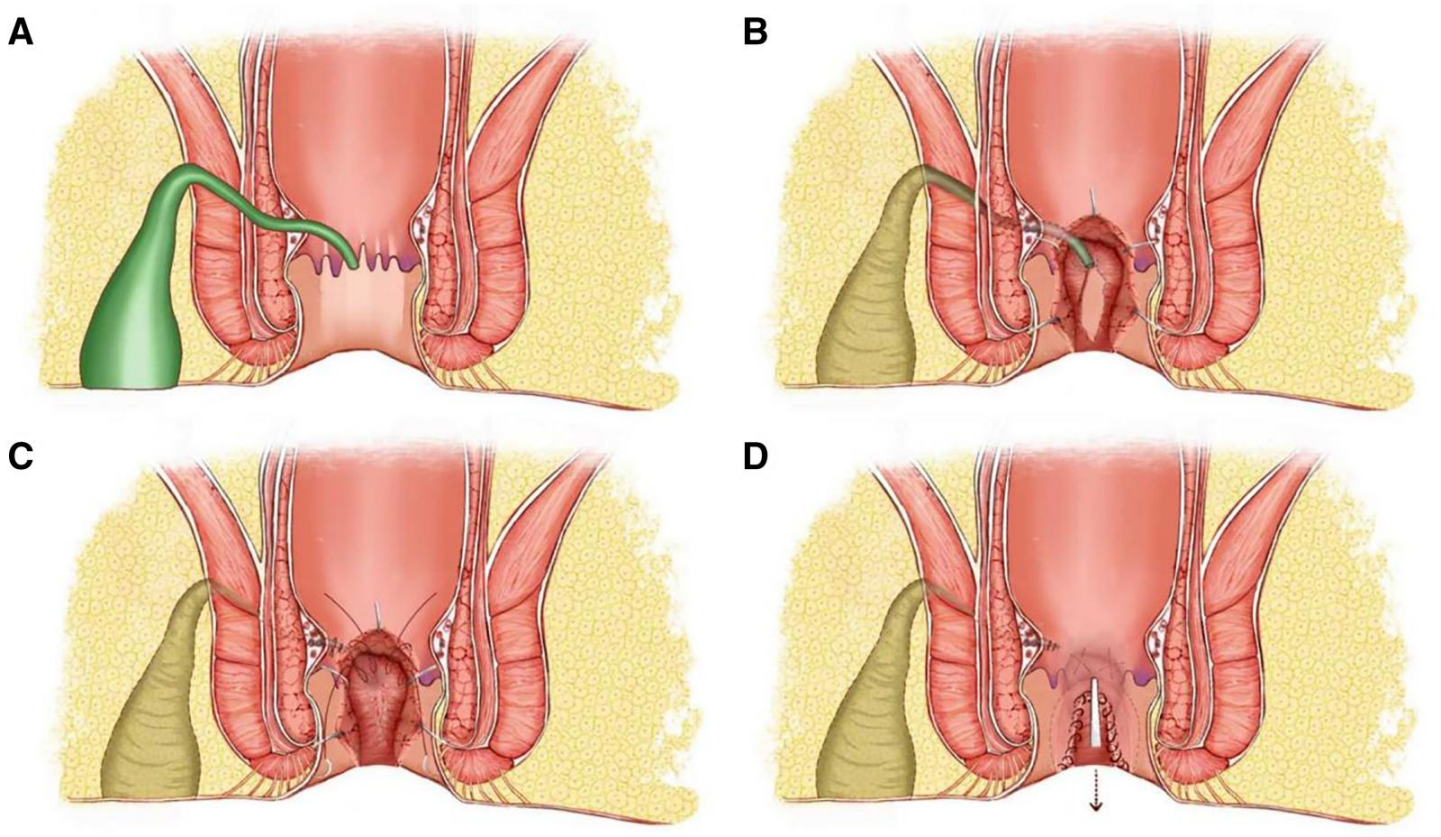
Surgical diagram of fistula occlusion with the internal sphincter flap. (A) A suprasphincteric anal fistula is identified with IO located at 6 o'clock. (B) A segmented processing technique consists of the fistulectomy in the ischiorectal fossa and ends at the lateral of the external sphincter, resection of IO and the internal sphincter underneath, and curettage of the fistula in the intersphincteric space and external sphincter. (C) Ligation or suture is conducted to close the tract that went through the external sphincter, and an internal sphincter flap is prepared for the coverage. (D) Flap occlusion is accomplished with continuous barbed suture, followed by the closure of the intersphincteric space. IO = internal opening.

A digital rectal examination, standard laboratory tests, and magnetic resonance imaging (MRI) were conducted on all patients. The classification was documented based on MRI findings, and the anal function was assessed by anorectal manometry and Wexner continence score [9]. The width of the fistula was measured as the diameter of the fistula passing through the external sphincter on axial T2-weighted images. The length of the fistula was measured by the distance from the internal orifice to the external sphincter perforation on coronal T2-weighted images. The height of the internal orifice is measured from the internal orifice to the anal margin on the sagittal T2-weighted image. Two radiologists with years of experience in diagnosing gastrointestinal diseases measured and averaged the results. Colonoscopy was performed when necessary to assess the condition of the intestine, ruling out conditions such as Crohn’s disease, ulcerative colitis, tuberculosis infection, and malignant tumors. All patients completed a consent form outlining the surgical procedure and received an enema the night before surgery. All patients were administered combined spinal-epidural anesthesia and placed in the prone position for surgery. The surgical diagram of FOISF is shown in Figure 1.

#### Details of technical processes of FOISF

##### Identifying the internal opening

**Figure 2.**
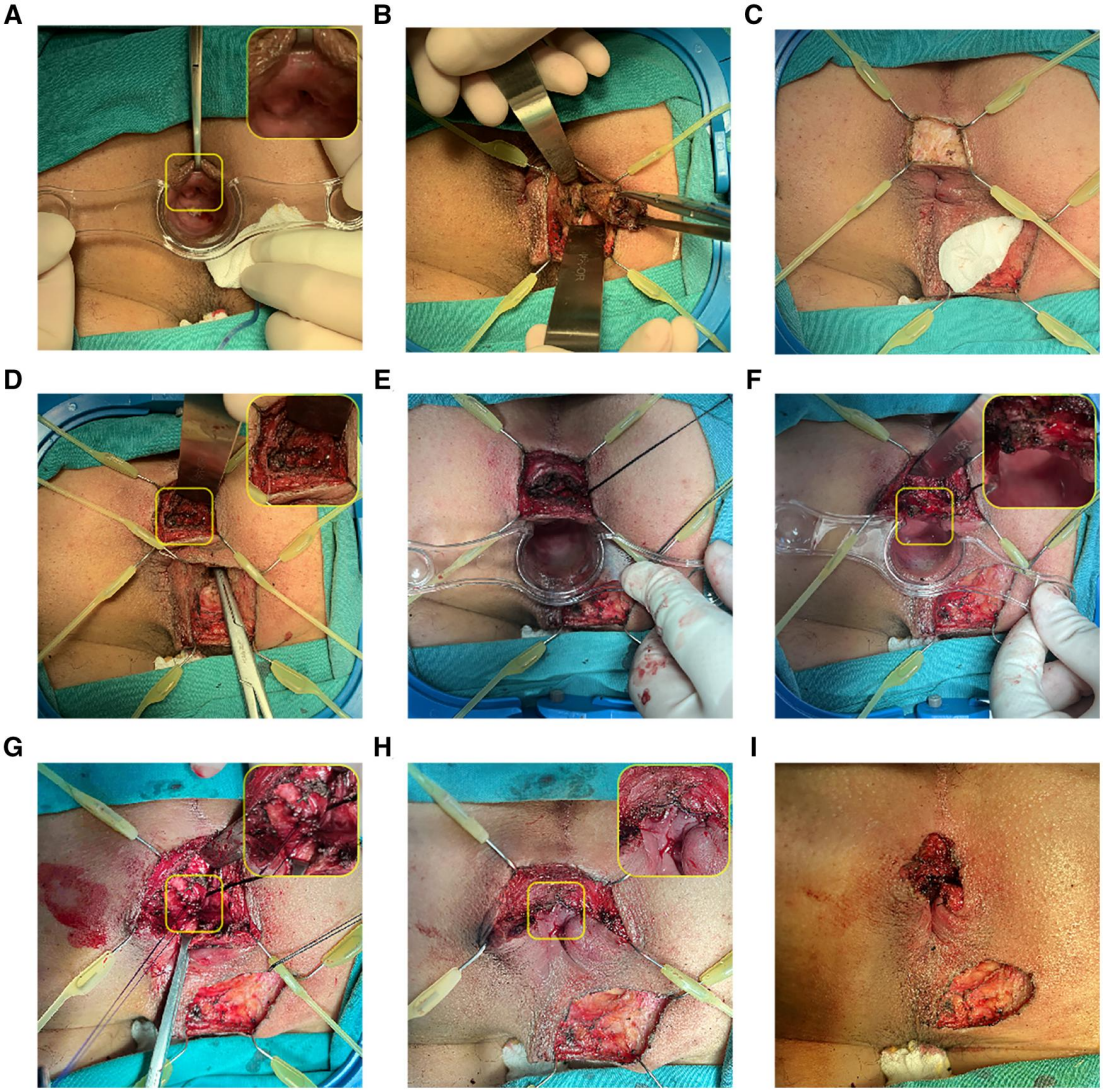
A posterior high transphincteric anal fistula was treated with fistula occlusion with the internal sphincter flap. (A) The IO located at 6 o'clock was identified by administering low-dose methylene blue with hydrogen peroxide. (B) A tunnel-like core fistulectomy was conducted from the superficial external opening into the deep ischiorectal fossa. (C) Intersphincteric incision was applied with a retractor at the corresponding anal clock of IO. (D) The portion of the tract that crossed the external sphincter was found, and the tip of the forceps is shown in the image. (E) Deep intersphincteric plane was fully laid open, followed by curettage. (F) The IO, together with the internal sphincter, was resected and incised separately. (G) Two intermittent stitches were applied in the intersphincteric space to sew the defect caused by the tract crossing the external sphincter. (H) The internal sphincter flap was further sealed or advanced up to the external sphincter to cover the stitches at the defect by using absorbable continuous barbed suture. (I) Completed surgery. IO = internal opening.

The most important first step is the identification of the IO. Typically, the IO was located by injecting a low dose of methylene blue with hydrogen peroxide through a superficial vein catheter from the external opening (Figure 2A). If the blue bubbles fail to emerge, gentle tract probing is an alternative. When the IOs could not be anchored by intraoperative methods in some cases, preoperative MRI findings were used for assistance.

##### Excision of the fistula from external opening to external anal sphincter

Next, lesions located outside the external anal sphincter (EAS) were treated by the principle of striated muscle protection. A tunnel-like core fistulectomy was performed from the superficial external opening into the ischiorectal fossa, ending at the entrance infixed into the EAS or puborectal muscle (Figure 2B). Transection of the terminal of the fistula at the outer layer of the EAS was recognized by the blue dye or probe. EAS was extracted by the Lone Star Retractor System and leaf retractor to avert possible damage. In case of supralevator lesions—accompanied by either fistula or abscess—excision or fenestration was followed by opening through the intersection angle of the levator ani muscle.

##### Dissection of the intersphincteric plane

A reversed T-shape incision was made at the corresponding anal clock of IO (Figure 2C). Then, a 2-cm-long arc incision was designed for better intersphincteric plane dissection and radial incision for later drainage of secondary wound healing. The Lone Star retractor system and leaf retractor were again applied for exposure and to lay open the fistula in the intersphincteric space. Once the foci in the intersphincteric plane were identified, forceps or a probe were used to locate the tract crossing the EAS (Figure 2D and E).

##### Resection of IO and closure of the transphincteric tract

The IO was resected, and the distal part of internal sphincter was laid open concurrently (Figure 2F). For the tract traversed from the inside of the external sphincter, we used 2-0 absorbable sutures with eight stitches to close the defect or ligate the tract alternately if the fistula wall was intact (Figure 2G).

##### Occlusion with the internal sphincter flap

An internal sphincteric flap comprising the mucosa, submucosa, and smooth muscle fibers was meticulously crafted, extending from the initial IO toward the cephalad. The flap was advanced to seal the transphincteric suture (at least 0.5 cm of flap for occlusion) and partially stitched at the external sphincter by absorbable continuous barbed sutures (Figure 2H and I). Perfect hemostasis was attained, and a rectal gauze pack was routinely inserted for hemostasis.

### Intraoperative and postoperative management

The operative time was defined as the period from the start of the surgery to the application of the dressing. The volume of blood loss was estimated by visual assessment, including tallying the number of sponges and gauze packs utilized and recording the suction containers. All patients received standard postoperative care, including 1-week oral antibiotics, fiber supplements and laxatives for constipation, warm site baths twice a day, and local dressing changes daily at the hospital for 2–3 weeks. The patients were discharged between Days 3 and 5 after surgery. Pain was assessed using the visual analog scale (VAS) with a range from 0 to 10 on Day 1 after surgery.

### Follow-up

Each patient was followed up for more than 6 months. Telephone interviews during the final visit were conducted to gather information, which included assessing routine clinical symptoms, evaluating anal continence using the Wexner continence score [9], and measuring quality of life using the Quality of Life in Patients with Anal Fistula Questionnaire (QoLAF-Q) [10]. The fistula was deemed healed only when no pus discharge was present, and all tracts had completely closed and healed; the presence of even a single tract with pus discharge was considered treatment failure. Recurrences were defined as the clinical or radiological confirmation of a new fistula’s emergence following the surgical procedure.

### Statistical analysis

All statistical analyses were conducted using SPSS version 26.0 (IBM Corporation, Armonk, NY, USA). Categorical variables were characterized using both numerical counts and percentages, while continuous variables were described using medians and interquartile ranges (IQR) or median (range). To assess significant differences in variables between groups, a *t*-test was used for quantitative data, while chi-square or Fisher’s exact test was used for categorical data. A significance level of *P *<* *0.05 was considered to indicate statistically significant differences.

## Results

### Patients’ characteristics

A total of 15 patients [13 male, median age (IQR): 33 (31, 42)] were included in this study. Detailed clinical and demographic characteristics of the patients can be found in Table 1. The median body mass index (BMI) of the subjects was 23.14 (22.04–25.90) kg/m^2^. Only one patient was diagnosed with diabetes mellitus, and five were identified as habitual smokers. Eight patients had a history of anal surgery, with one person undergoing the procedure twice. All patients had transphincteric or suprasphincteric anal fistulas, with eight having a combined horseshoe-like fistula and eight experiencing an associated abscess. Three types of horseshoe-like extension courses were observed, including four intersphincteric-ischioanal horseshoe-like fistulas, three intersphincteric horseshoe-like fistulas, and one classical ischioanal horseshoe-like fistula. In addition, four locations of associated abscesses were identified, namely ischiorectal fossa (*n *=* *4), intersphincteric region (*n *=* *2), inter-levator ani muscle (*n *=* *1), and intersphincteric-ischioanal abscesses (*n *=* *1). In total, there were 11 posterior IO, three lateral IO, and one anterior IO, with the median (IQR) height of IO measuring 2.3 (2.1–3.25) cm. Furthermore, within the study group, it is worth noting that 11 patients presented with multiple tracts. The median (IQR) width of the tract crossing the external sphincter was 0.8 (0.5–1.05) cm. Most patients exhibited complex intersphincteric extension, with the median (IQR) length measuring 3 (1.55–3.4) cm.

**Table 1.** Clinical and demographic characteristics of the patients

Patients	Age, years	Gender	Smoking	Comorbidities	History of anal surgery	Park’s classification	Horseshoe-like fistula	Associated abscess	Internal opening location	Internal opening height, cm	Multiple tracts	The width of the tract crossing the external sphincter, cm	The length of the intersphincteric fistulous tract, cm	The interoperative methylene blue and hydrogen
1	30	Male	Yes	None	Yes	High transsphincteric	None	Ischioanal	6	2.2	None	0.5	1.1	N/A
2	30	Female	Yes	None	None	Low transsphincteric	Intersphincteric	None	12	1.5	Intersphincteric horseshoe-like fistula	0.4	3.1	N/A
3	36	Male	Yes	None	None	High transsphincteric	None	None	6	2.1	None	0.4	0.6	Blue-dyed
4	31	Female	None	None	None	Low transsphincteric + high intersphincteric	Intersphincteric	Ischioanal	7	2.3	Intersphincteric horseshoe-like fistula + rectal intermuscular	0.5	3.9	Blue-dyed
5	31	Male	None	None	None	High transsphincteric	Intersphincteric-ischioanal	Ischioanal	6	3.4	Intersphincteric horseshoe-like fistula	0.5	3.2	Blue-dyed
6	33	Male	None	None	Yes	Suprasphincteric	None	None	6	3.7	Supra-levator fistula	0.8	5.4	N/A
7	28	Male	None	None	Yes	High transsphincteric	Classical ischioanal	Intersphincteric	6	2.1	Ischioanal	1.2	3.0	Blue-dyed
8	32	Male	None	None	None	High transsphincteric	None	Intersphincteric	6	3.6	None	1.0	2.3	Blue-dyed
9	66	Male	None	None	Yes	High transsphincteric	None	Intersphincteric-ischioanal	6	2.0	Ischioanal fistula	1.1	1.0	N/A
10	40	Male	None	None	Yes	Low transsphincteric	None	None	6	2.3	None	0.6	1.0	Blue-dyed
11	44	Male	Yes	None	None	High transsphincteric	Intersphincteric-ischioanal	None	7	3.1	High intersphincteric-ischioanal horseshoe-like fistula	0.8	3.4	N/A
12	40	Male	None	None	None	Suprasphincteric	Intersphincteric-ischioanal	None	6	2.9	Inter-puborectalis muscle horseshoe-like fistula	0.7	2.6	N/A
13	48	Male	Yes	None	Yes	Suprasphincteric	None	Inter-levator	7	3.5	Inter-levator ani muscular	1.4	4.5	N/A
14	68	Male	None	Diabetes mellitus	Yes	Suprasphincteric	Intersphincteric	None	6	1.9	Inter-levator ani muscular + ischioanal	0.8	2.0	Blue-dyed
15	33	Male	None	None	Yes	High transsphincteric	Intersphincteric-ischioanal	Ischioanal	6	2.5	Inter-puborectal muscle horseshoe-like fistula	1.4	3.4	N/A

N/A = methylene blue staining was negative.

### Post-surgical outcomes of patients

All patients underwent the surgical procedure, with a median (IQR) operation time of 53 (41–47) min. The median (IQR) volume of blood loss during the surgical intervention was 13 (10–16) mL. No unexpected adverse events occurred during the surgical procedure. All patients completed the Wexner scores, VAS scores, and QoLAF-Q assessments. We evaluated postoperative pain using VAS scores, with median (IQR) scores of 8 (7–9.5), 4 (3–5.25), and 1 (1–2.25) at 1 week, 1 month, and 3 months after the procedure, respectively. Fourteen patients achieved primary intention healing. Only one patient was diagnosed with rupture of flap and unclosed transphincteric tract on postoperative day 18. He was encouraged to flush saline daily with an infusion tube from the wound of the external opening; the internal flap finally healed by second intention by postoperative day 40. Over a follow-up period of at least 11 months [median: 20 (14–30) months], no recurrence was observed. Importantly, the patients exhibited satisfactory anal continence, with no statistically significant difference observed between the preoperative and postoperative Wexner scores (*P *=* *0.331; Table 2). Moreover, a significant improvement in the quality of life compared with the preoperative assessment was observed (*P *<* *0.001). Finally, anorectal manometry assessments were conducted both preoperatively and during the last follow-up postoperatively. The findings revealed minimal differences with respect to maximum squeeze pressure, average resting pressure, and the high-pressure zone between the preoperative and postoperative phases (*P *=* *0.268, 0.504, and 0.343, respectively).

**Table 2.** Functional outcomes of patients with complex anal fistula

Variable	Pre-operation	Post-operation	*P*-value
Wexner score, median (range)	1 (0–2)	1 (0–4)	0.331
QoLAF-Q, median (range)	32 (21–51)	21 (19–29)	<0.001
Maximum squeeze pressure, mmHg, median (range)	164.7 (83.4–344.4)	134.9 (79.9–294.2)	0.268
Average resting pressure, mmHg, median (range)	74.8 (50.0–105.4)	75.9 (50.5–108.2)	0.504
High pressure zone, cm, median (range)	4.2 (2.9–5.6)	4.0 (2.7–4.8)	0.343

QoLAF-Q = Quality of Life in Patients with Anal Fistula Questionnaire.

## Discussion

The sphincter-preserving technique is an ideal surgical intervention for complex anal fistulas. However, no method has yet been demonstrated to be perfectly successful in treating high complex anal fistula. The FOISF method described in this study can destroy the infected crypt glands and protect anal function. Based on preliminary results, FOISF appears to be a safe and effective treatment for high complex anal fistulas.

Among the sphincter-sparing procedures, LIFT, TROPIS, and ERAF are the mainstream techniques but have different ways of processing the intersphincteric foci based on their own theory foundation. In 2007, Rojanasakul [6] was the first to introduce LIFT to focus on ligation of the intrasphincteric fistula tract but not on resection or drainage, which exhibited amazing outcomes with a 94.4% success rate. However, the subsequent studies [11–13] failed to show a cure rate as high as Rojanasakul’s, or even less than half. In light of this issue, several researchers have hypothesized that while LIFT may excise the intersphincteric infection, it may leave behind fibrosis and secretion residues or incompletely ligate the fistula tract, particularly in cases of high-complexity fistulas [14]. Besides, meticulous dissection for the intact inter-sphincteric fistulas and foolproof ligation is not always easy to perform, even for a sophisticated proctologist [11]. As such, LIFT may not be recommended for anal fistulas with high intersphincteric foci [14]. However, TROPIS seems more suitable for high complex anal fistula, as seen in two large-sample, multicentric studies [15, 16]. Garg, who first described the TROPIS technique, emphasized the preservation of the external sphincter through the methods of curettage and tract cleaning, as well as tube insertion. Moreover, the intersphincteric portion is managed postoperatively through continuous drainage by deroofing [7]. Meticulous postoperative care is required, especially for the first 2 weeks [17]. It is risky that the transphincteric portion remains unhealed and is being infused with infectious bacteria. Finally, ERAF, as described by Elting [18] in 1912, has a long history in the treatment of anal fistula as an optional technique. However, ERAF also exhibits a variable failure rate, ranging from 10% to 60% [19–24]. Potential causes of failure following the ERAF procedure may primarily involve compromised blood flow because of tension or a too-narrow base of the flap, resulting in devascularization, along with overlooked extensions with inadequate drainage or local infection beneath the flap. With the needs of minimally invasive technique and rapid recovery, video-assisted anal fistula treatment and over-the-scope clip have gradually been accepted as sphincter-saving alternatives. Unfortunately, incomplete closure of IO, as well as the undrained infection, remain the main reason for surgical failure [25, 26].

**Figure 3.**
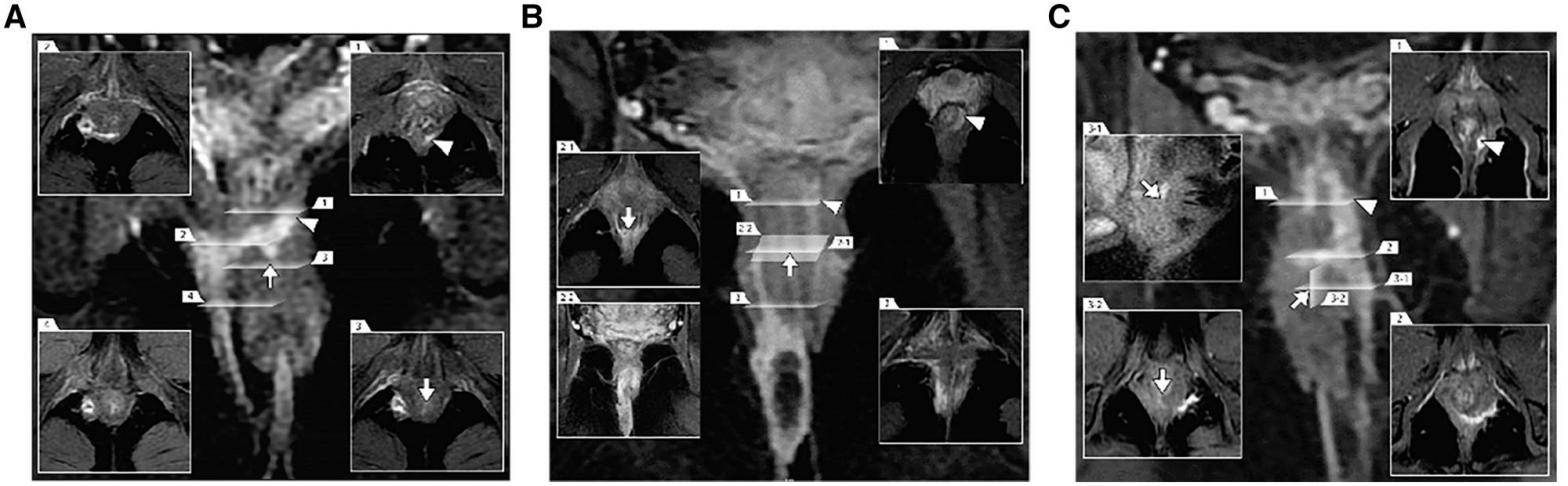
Different types of posterior complex anal fistulas from our cohort, as seen on coronal contrast-enhanced fat-suppressed T1-weighted images with different corresponding sections. (A) The suprasphincteric anal fistula with multiple branches in the deep intersphincteric horseshoe tracts and right ischiorectal fossa. (B) The low transphincteric anal fistula with right ischiorectal fossa abscess and deep intersphincteric horseshoe tracts, extending along the left combined longitudinal muscle into the supralevator area of the rectal muscle. (C) The high transsphincteric anal fistula in the left puborectal muscle with up spread supralevator into the pelvirectal space. The arrows are pointing at the IO. The triangles represent deep branches. IO = internal opening.

With FOISF, it is noteworthy that all 15 cases (100%) achieved complete healing, with no adverse event reported. Our patients mainly presented with high-complexity anal fistulas, characterized by multiple tracts, associated abscesses, and high-positioned horse-like extension tracts in the intersphincteric space, posing challenges for effective management. Figure 3A–C depicts three types of complex anal fistulas, each presenting significant challenges. Figure 3A illustrates a suprasphincteric anal fistula with multiple branches, while Figure 3B exhibits a low transphincteric anal fistula with an abscess and horseshoe tracts extending into the supralevator area of the rectal muscle. Figure 3C shows a high transsphincteric anal fistula with upward extension into the supralevator and pelvirectal space. The LIFT procedure is not recommended in our cohort as it may pose challenges in completely removing the high intersphincteric foci. Furthermore, it needs a bigger intersphincteric groove incision to ensure the ligation of high tracts. A randomized controlled trial by Li *et al.* [27] focused on complex anal fistulas and reported a success rate of only 42%. Additionally, TROPIS presents challenges in addressing the complex extension of tracts in the intersphincteric space, given the limited incision extending only up to the IO and leaving untreated tracts that cross the external sphincter. This limitation may result in incomplete treatment of the tracts, leading to possible recurrence. In the new approach, we resected the IO together with the internal sphincter. The FOISF allows for easy exposure of the intersphincteric space and enables complete treatment of the branches within it and the closure of tracts that cross the external sphincter.

In this study, we employed a partial advancement flap technique to cover the sutured external sphincter [28]. This flap shows a lower tension than traditional ERAF, which greatly reduces the probability of flap tearing. We could seal the flap from the cephalic endorectal to the caudal side, or from the lateral side to the middle, depending on the intersphincteric branches and the pattern of the tract crossing the external sphincter. The cephalic flap is applied for multi-tracts or horseshoe tracts in the intersphincteric space, while the lateral flap is perfectly suitable for the unique side transphincteric tract. This flap allowed us to mitigate the risk of potential anal infections and reduce the likelihood of recurrence.

**Figure 4.**
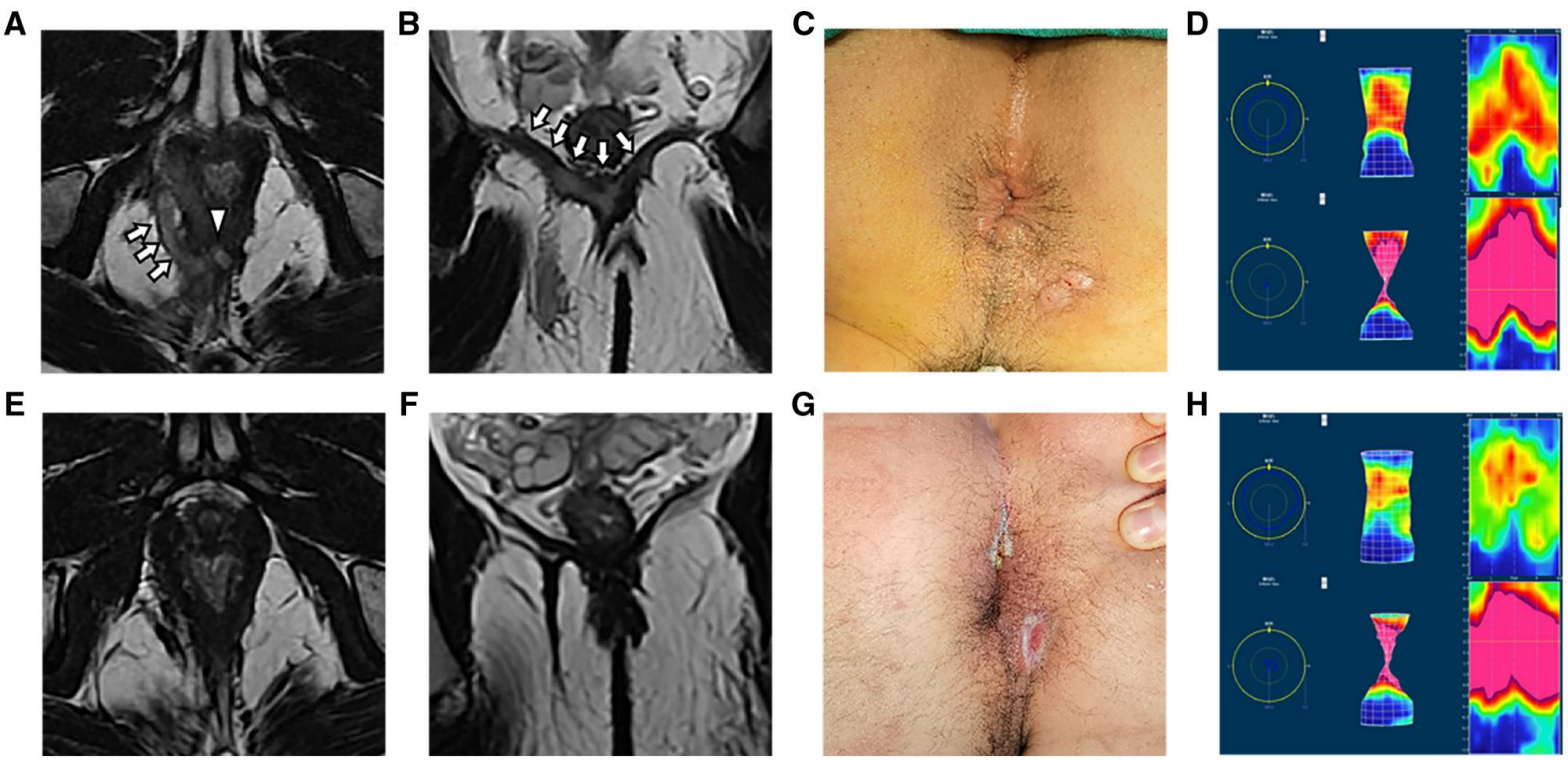
A high transsphincteric anal fistula with abscess in the right ischiorectal fossa and bilateral intra-levator ani muscle were treated by fistula occlusion with the internal sphincter flap with complete healing on follow-up. Preoperative and postoperative magnetic resonance imaging scans were performed to verify the complexity of the tract and internal opening and recovery by (A, E) axial T2-weighted images and (B, F) coronal T2-weighted images. (C, G) Photograph of the anus shown preoperatively and at the last follow-up. (D, H) The anorectal manometry assessments were carried out preoperatively and at the last follow-up. The arrows are pointing at the tracts. The triangle represents the IO. IO = internal opening.

Furthermore, it is important to highlight that there were no significant differences in anal function when compared with the preoperative condition. The assessment of anal function using the Wexner score and anorectal manometry revealed no significant differences (Table 2). Figure 4 is presenting a typical case with high transsphincteric anal fistula treated by FOISF, where we can find a normal anal appearance and unblemished manometry result postoperatively. FOISF technique, as well as TROPIS, inevitably sacrifices partial internal sphincter to ensure the cure rate and protect the anal function [7, 15]. It’s widely known that fistulectomy with internal sphincter resection is efficacious in low anal fistulas and with gratifying anal function [29, 30].

The small cohort size and the retrospective nature of the analysis are some of the limitations of this study. However, FOISF for treatment of high complex anal fistula exhibited a promotive outcome.

## Conclusions

Our preliminary results present an effective approach to complex transphincteric anal fistula with a high healing rate, minimal pain, rapid return to normal activities, and satisfactory anal continence and function. We recommend conducting larger prospective studies to further validate these findings.

## Authors’ Contributions

Z.L. and D.R. served as the principal investigators responsible for the study plan; D.H., Yiping L., and N.W. participated in drafting the manuscript; K.W. and Y.Y. collected the data; Z.L. and Yanzhu L. were responsible for finalizing the manuscript. All authors have read and approved the final version of the manuscript.
